# Effect of weathered on the pore characteristics of different rocks in coal-bearing strata

**DOI:** 10.1371/journal.pone.0320446

**Published:** 2025-03-28

**Authors:** Dong Feng, Enke Hou, Xiaoshen Xie, Pengfei Hou, Jiangbo Wei

**Affiliations:** 1 College of Geology and Environment, Xi’an University of Science and Technology, Xi’an, Shaanxi, China; 2 Shaanxi Provincial Key Laboratory of Geological Support for Coal Green Exploitation, Xi’an, Shaanxi, China; Hefei University of Technology School of Resources and Environmental Engineering, CHINA

## Abstract

This study aims to explore how weathered controls the microscopic pore development of rocks. Siltstones to coarse sandstones were selected from the main rocks of the Jurassic strata of the northern Ordos Basin. The pore structure, mineral composition, the pore size distribution (PSD), porosity, permeability and fractal characteristics of unweathered and weathered rocks were analysed using a combination of X-ray diffraction (XRD), scanning electron microscopy (SEM) and low-field nuclear magnetic resonance (LF-NMR). The study showed that the microstructure of different sandstones has obvious differences, the total porosity and permeability of rocks are usually positively correlated with the grain size of the rock, and the microscopic pore structure of rocks of the same lithology changes from small pore type to large pore type after weathered. The results show that weathered rocks had the same mineral composition as unweathered rocks specimens but different intensity peaks. It can be clearly seen that the intensity of clay minerals significantly increased in the weathered rock. whereas the proportion of quartz and feldspar decreases correspondingly. The proportion of clay minerals present in the sandstone increases from 4.75% to 13.25% as a consequence of weathered. The sandstone, which exhibited a greater proportion of micropores and fine pores prior to weathered, demonstrated a higher prevalence of macropores and a corresponding increase in fractal dimensions following weathered. The fractal dimensions D_1_, D_2_, and D_T_ of the various lithologies exhibited an overall decreasing trend with an increase in particle size. It should be noted that a rock with the same fractal dimension or porosity may exhibit different microporous structures. The research results play an important theoretical support for the prediction of the degree of water enrichment when encountering the weathered sandstone in the process of coal mining, and have an important theoretical significance for the prevention of water hazards that may be encountered in the process of coal mining.

## 1 Introduction

The aquifers in Jurassic coal fields in the northern Ordos Basin, China, are controlled by the stratigraphic sedimentary conditions. Aquifers vary greatly in the spatial characteristics of lithology, petrography, and water-richness, especially the pore structures of different rocks [1,2]. Pore structure differences are one of the main factors leading to the uneven fugacity of aquifers, and pore structure differences in different rocks are the link between the depositional environment and fugacity [3,4]. Therefore, testing and analytical evaluation to reveal the commonalities and differences in the microporous characteristics of weathered and corresponding unweathered rock masses of different lithologies can better provide a reference basis for the safe mining of coal. Several traditional and and new methods have been used to test the size and connectivity of microscopic pores within rocks [5–7]. For instance, Optical and scanning electron microscopes [8,9], low-temperature nitrogen adsorption [10,11], and mercury-pressure experiments have been widely used to measure the pore structures of porous media [12,13]. However, these techniques have some limitations, probably leading to interference during the experiments. Scanning electron microscopy can only qualitatively characterize pore morphology at the micron scale but cannot quantitatively evaluate nanopores. In mercury-pressure experiments, artificial damage to specimens may be caused, and 100% saturation cannot be guaranteed when measuring the porosity [14–16]. Low-temperature nitrogen adsorption has limitations on pore size testing [17].

**Fig 1:**
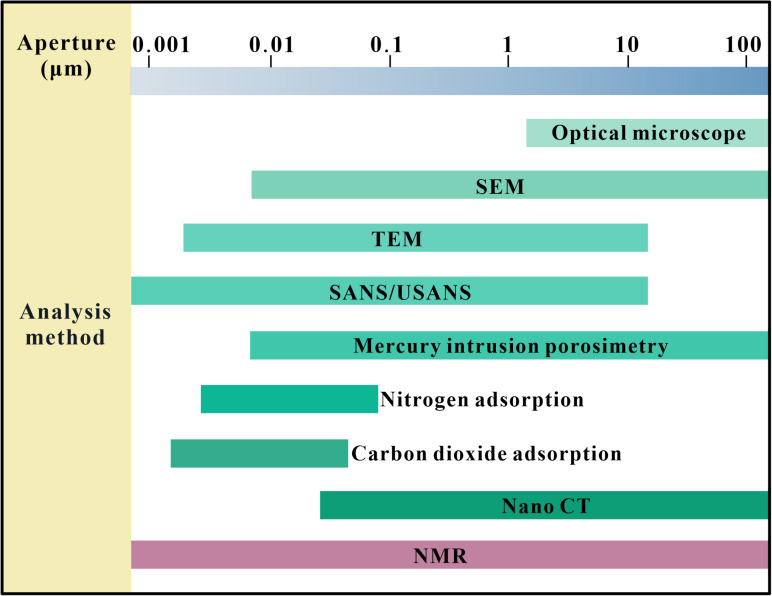
Classification of rock pores and methods of study.

LF-NMR is a non-invasive, real-time monitoring experimental method for the assessment and analysis of the microscopic pore structure of materials and has a wide range of applications (Fig 1). It is now widely used to assess the microscopic pore characteristics of porous media. The results obtained from LF-NMR analysis are highly comparable to those obtained from low-temperature nitrogen adsorption and mercury compression tests. This method makes up for the shortcomings of other test methods and is more accurate and convenient. It is fast and informative with a wide range of measurements and does not damage the internal space of rocks [18,19]. At present, LF-NMR has now proven to be one of the most effective techniques for characterising reservoir pore properties, such as capturing the pore size distribution, permeability and pore fluid connectivity of different micropores [20–22].

The depositional process of the rock formation has resulted in a relatively complex pore structure and physical properties, with diagenesis controlling the pore structure, porosity and permeability of the rock mass [23–25].

Among them, fractal theory is a mathematical method used to describe the structural complexity and to quantitatively characterise the complexity of the pore structure of rocks [26,27]. Since the creation of fractal theory by Mandelbrot in 1980, it has been widely applied in describing the analysis of physical properties of natural porous media, such as permeability, microscopic pore structure and inhomogeneity [28]. However, the traditional single fractal approach is unable to characterise complex porous media with significant pore differences but the same fractal dimension [29]. In recent years, in order to characterise the fractal differences of complex porous media in more detail, researchers have introduced the multiple fractal theory to analyse and characterise the pore structure of rock bodies [30,31]. Multiple fractal theory can better describe the complex microstructure of low-permeability reservoirs, and can use parameters such as fractal dimension to quantitatively characterise the complexity of the microscopic pore mechanism, which is simple to compute and highly applicable.

Nevertheless, much of the previous researches have mostly focused on focused on the analysis of microscopic pore characteristics of single rocks, and there are few studies on the commonalities and differences of the pore characteristics of different rocks [32,33]. In particular, the effects of weathered on the pore characteristics of different rocks are not considered. Whether the effects of weathered on the porosity, pore types, and connectivity of rocks with different grain sizes remain constant is still unclear, and further relevant studies are necessary.

In this study, LF-NMR was used to measure the microscopic porosity characteristics of different rocks with a view to addressing two main questions. (1) What are the differences in the porosity, pore types, pore size percentages, and permeability of different rocks? (2) What are the differences in the effects of weathered on the pore structures of different rocks in coal-bearing strata?

## 2 Materials and methods

### 2.1 Study site and samples

**Fig 2:**
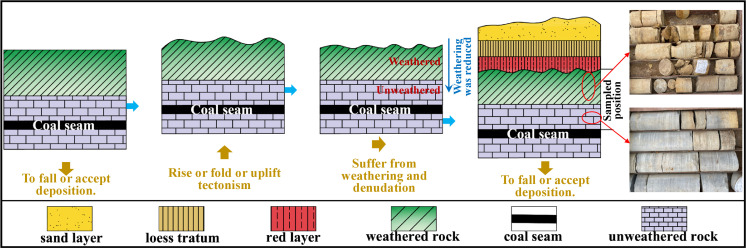
Sampling location diagram.

The Ordos is a multi-cyclonic Craton basin, covering a total area of 250 000 × 104 km^2^. The local coal resources represent a quarter of the total volume in China. The roof strata of the coal seam primarily consist of mudstone, sandstone, and coal layer. Under the historically geological effect, the rising earth’s crusts or the tectonic processes (such as folding and uplifting) disturb the deposited coal-bearing strata and remove parts of the overlying bedrock. Consequently, these strata experience weathering and spalling. Due to these events, the resulting spatial behaviour is characterised by the obvious vertical upward banding. In addition, the influence of weathering reduces when the depth increases, resulting in a transition from weathered to unweathered layers. Weathering decreases the cementation degree of the rock layer and increases its porosity. As a result, the weathered rock layer possesses better permeability and water storage conditions than the unweathered rock. With these properties, it becomes the most important aquifer in coal seam mining and the primary source of underground water (Fig 2).

Combined with the drill hole coring in the study area, this study qualitatively classified the weathered bedrock and unweathered bedrock in the study area mainly based on the colour, structure and degree of fragmentation of the rocks, and the specific classification criteria and characteristics are shown in Table 1.

**Table 1:** Comparative classification and characterisation of weathered levels.

Weathered or not	Colour	Structural characteristic	Fragmentation degree
Unweathered	Gray	Long columnar	The rockcore integrity is high.
Weathered	Light grayish-yellow, light yellow-green	Lumpy, lamellar structure, long columnar, the structure is slightly damaged, relatively complete	Slightly broken, few weathered cracks

The rock samples used in this thesis test were taken from the Jurassic Yan’an Formation stratum of Hongliulin Coal Mine in the north of Yulin, Shaanxi Province, and were mainly selected from siltstones, fine sandstones, medium sandstones and coarse sandstones of the unweathered and weathered rock layers at the top plate of the coal seam, and the depth of the samples taken was about 43.55 m~125.95 m. Among them, the unweathered sandstones were generally gray, and most of them became yellow and yellow-green after weathered. In order to ensure the high accuracy of the experimental results, the random system sampling method was adopted, and 4 samples were extracted in parallel from each group, and a total of 32 groups of specimen were prepared. Each specimen was a cylinder of Φ 25 mm × 50 mm. The environment during sample preparation and sampling was room temperature of 27° and humidity of 30%, the mass error of each group was within ±0.41%, the particle size of parallel samples was within the same range, and the specific surface area of all the samples was 0.004 m^2^. The rock samples were numbered according to their lithology types and whether weathered occurred. Table 2 shows the rock type and degree of weathered of this study.

**Table 2:** Rock physical parameters of the samples.

Rock type	Sample	Weathered or not	Rock type	Sample	Weathered or not
Coarse sandstone	C1	Unweathered	Medium sandstone	M1	Unweathered
	C2	Unweathered		M2	Unweathered
	C3	Unweathered		M3	Unweathered
	C4	Unweathered		M4	Unweathered
	C5	Weathered		M5	Weathered
	C6	Weathered		M6	Weathered
	C7	Weathered		M7	Weathered
	C8	Weathered		M8	Weathered
Fine sandstone	F1	Unweathered	Siltstone	S1	Unweathered
	F2	Unweathered		S2	Unweathered
	F3	Unweathered		S3	Unweathered
	F4	Unweathered		S4	Unweathered
	F5	Weathered		S5	Weathered
	F6	Weathered		S6	Weathered
	F7	Weathered		S7	Weathered
	F8	Weathered		S8	Weathered

### 2.2 Experimental procedure

**Fig. 3:**
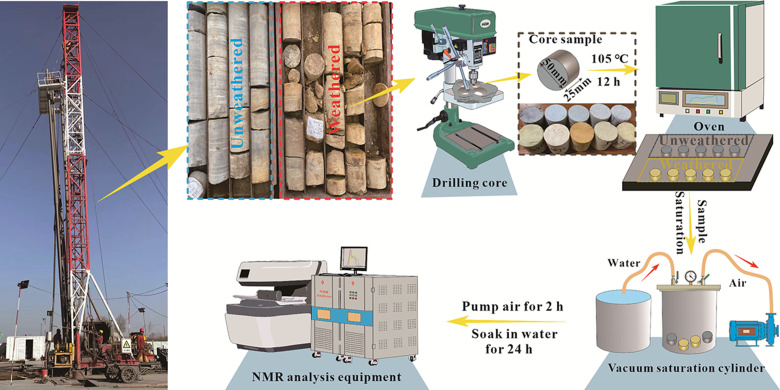
Experimental flow chart.

The artificially produced rock samples were placed inside a drying chamber and subjected to a temperature of 105 °C for about 12 hours, until their weight remained constant. Therefore, after the samples were dried, they were saturated in a vacuum saturation device with a vacuum pressure of -0.1 MPa for 24 h to reach the fully saturated state (Sw). LF-NMR was tested using MacroMR12-150H-1 spectrometer produced by China, Suzhou Niumag Analytical Instrument Corporation. Magnet type: permanent magnet; The magnetic field intensity was 0.3± 0.05t, the main frequency of the instrument was 12.8MHz, the measured nucleus was hydrogen atom, no solvent signal suppression was used during the experiment, and the experimental ambient temperature was 26°. Fig 3 shows the specific experimental flow chart.

### 2.3 Theory of LF-NMR testing

**Fig. 4:**
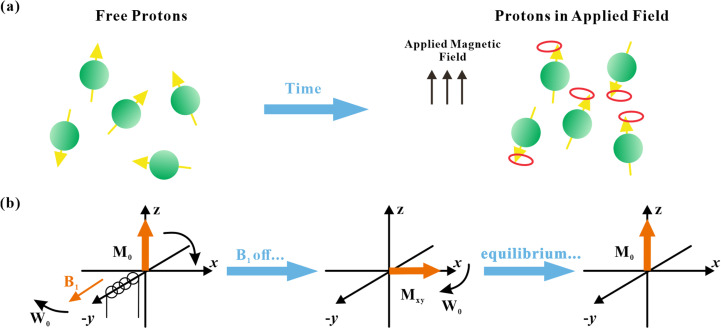
Principle of MNR. **(a)** Hydrogen atoms in the application domain; (b) changes in magnetization vectors under the action of the radio-frequency pulse.

Fig 4 shows the principle of LF-NMR. After obtaining T_2_ spectra in porous media by LF-NMR detection, a relationship between T_2_ and microporous pore size was established. Typically, a smaller relaxation time T_2_ indicates the microporous pore size [34].

The T_2_ spectra in the porous medium can be obtained using Eq (1). Specifically, the peak position and peak area are related to the number of different pore sizes [35,36].


$$\frac{1}{T_{2}}=\frac{1}{T_{2s}}\text{=}\rho \times \frac{S}{V}=\rho \times \frac{\omega }{r}$$ (1)

The formula takes into account several parameters such as *T*_2s_ (surface relaxation time), *ρ* (transverse surface relaxation strength coefficient of the rock), *S* (pore surface area), *V* (pore volume), *ω* (pore shape factor), and *r* (pore radius measured in micrometers).

Therefore, the rich pore information can be reflected by converting Eq (1) to different pore radius sizes.


$$r\text{=}\rho \times \omega \times T_{2}$$ (2)

The *T*_2_ of LF-NMR of each sample can be converted into the corresponding total porosity using Eq (3) by calibrating with a standard fluid-saturated sample with the given porosity.


$$\varphi_{nmr}(\%)=\sum_{i}\frac{m_{i}}{M_{b}}\times \frac{S_{b}}{s}\times \frac{G_{b}}{g}\times \frac{V_{b}}{V}\times 100\text{\%}$$ (3)

where *φ*_nmr_ denotes the porosity, m_i_ is the T_2_ amplitude of the ith T_2_ component, M_b_ is the total T_2_ amplitude of the sample, S_b_ and s are the number of scans of the standard and rock samples, G_b_ and g are the acceptance gains of the standard and rock samples, V_b_ and V are the water content volumes (cm^3^) of the standard and rock samples, respectively.

## 3 Results and discussion

### 3.1 Pore characteristics and mineral composition of sandstone

#### 3.1.1 SEM observation.

**Fig 5:**
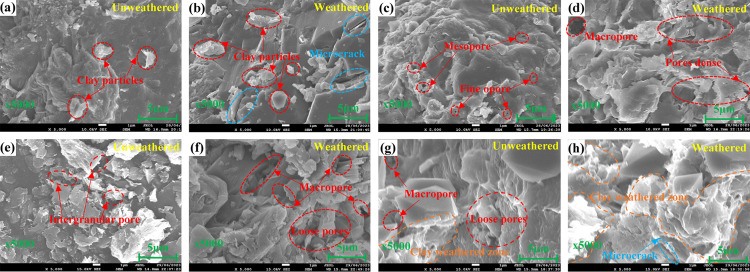
Electron microscope scanning of different sandstone samples. (a, b) Siltstone; (c, d) fine sandstone; (e, f) medium sandstone; (g, h) coarse sandstone.

Scanning electron microscopy (SEM) images (magnification *3000) of the unweathered and weathered sandstone are indicated in Fig 5. Fig 5a and b shows that the unweathered siltstone exhibits a dense structure, poorly-defined boundaries between particles, undetectable pore distribution, and limited development of clay mineral particles. After being weathered, microcracks develop intermittently, and dissolution pores and lamellar clay minerals appear between particles. Notably, most of these pores are small in diameter, and a limited number of macroporous pores are distributed locally, resulting in predominantly small pore sizes. Despite the presence of large pores formed by weathering, the total volume of voids remains relatively small. By comparing Fig 5c and d, it can be seen that fine sandstone and siltstone undergo similar microstructural changes due to the influence of weathering degree. A comparison between Fig 5e and f reveals that sandstone particles exhibit relatively uniform distribution and inter-granular pores; in contrast, stripping occurs in some smaller particles in the rock subjected to weathering, reducing the degree of contact between larger particles. Consequently, pore density and connectivity increase. According to the comparison of Fig 5g and h, the boundary of the unweathered coarse sandstone particles is obvious, the pores are compactly and uniformly distributed, and a concentrated, large-scale pore space is observed in the localized area; the number and density of pore spaces increase greatly under weathering, especially the scope of the clay weathered zone.

#### 3.1.2 Mineral composition testing.

**Fig 6:**
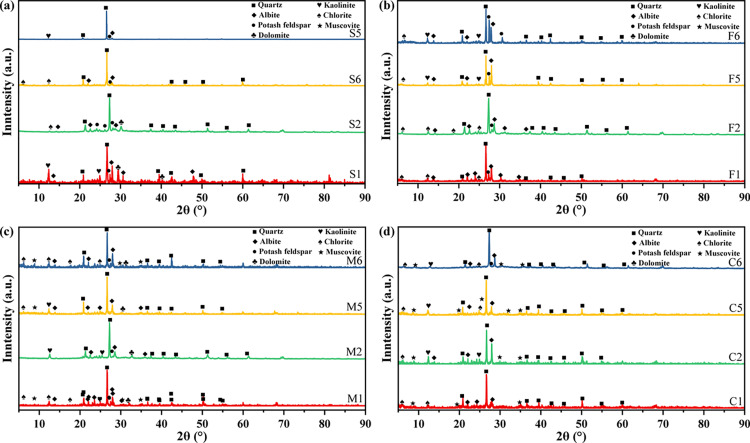
XRD of unweathered and weathered sandstones. (a) Siltstone; (b) fine sandstone; (c) medium sandstone; (d) coarse sandstone.

Fig 6 shows the XRD of different types of unweathered sandstone and weathered sandstone. Compared with the standard diffraction cards, the XRD of sandstone samples with different weathering degrees is basically consistent. This observation indicates that no changes occur in the main minerals in sandstone with different grain sizes due to weathering. To be specific, the ‘hard body’ part is primarily composed of detrital minerals such as quartz, sodium feldspar, potassium feldspar, and dolomite. These components constitute the basic structure of different sandstones and contribute to their strength. A higher quartz content results in a better compression resistance of the rock. In contrast, the ‘soft body’ mainly contains clay minerals, including kaolinite, chlorite and dolomite. This indicates that the mineral composition of the two parts varies depending on the degree of weathering.

**Fig 7:**
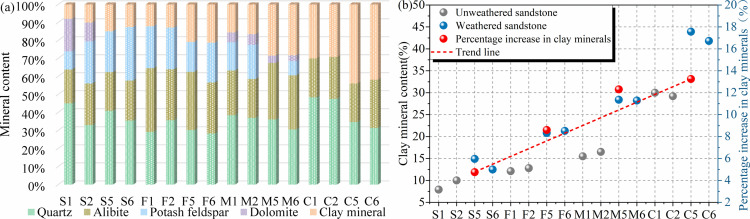
Changes in mineral composition of unweathered and weathered sandstones. (a) Percentage of mineral composition; (b) ratio of increase in clay minerals.

Fig 7 shows the percentage of mineral composition and the increase ratio of clay mineral content of different types of sandstones before and after weathered. Due to the different types of sandstone, the difference in mineral composition content is large, but the variation rules are the same after weathered. As can be seen from the figure, the increase of clay mineral content of sandstones after weathered ranges from 4.75% to 13.25%. Among them, the increase of clay mineral content of coarse sandstone after weathered is the largest, which reaches 13.25%. Overall, sandstones are subjected to weathered with a gradual decrease in quartz and feldspar and a gradual increase in clay minerals.

#### 3.1.3 Pore characteristics of LF-NMR.

The classification of pore size proposed by Hodot divides pores into three categories: small pores (< 0.1 μm), mesopores (0.1–1.0 μm) and macropores (> 1.0 μm) [37]. In recent years, researchers and scholars in order to more finely distinguish the trend of changes in the pore size of different sizes of pores, taking 0.01 μm as the division boundary, small pores can be further divided into microporous pores (< 0.01 μm) and fine pores (0.01–0.1 μm) [38,39]. Therefore, in this study, in order to better compare and analyse the trend of sandstone pore, pore sizes were classified into four categories, including micropores with pore size < 0.01 μm, fine pores with pore size 0.01-0.1 μm, mesopores with pore size 0.1-1.0 μm and macropores with pore size > 1.0 μm.

**Fig 8:**
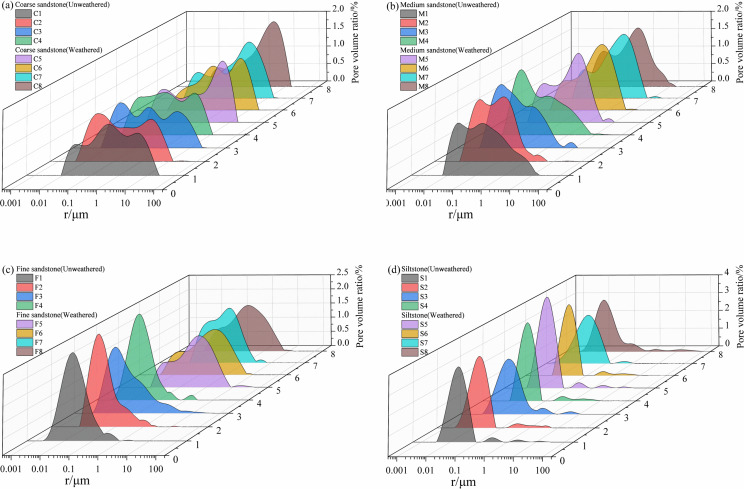
Pore size distribution of different sandstone. (a) **Coarse sandstone;** (b) **medium sandstone; (c) fine sandstone;** (d) **siltstone.**

Fig 8 shows the pore size distribution (PSD) of the different lithologies tested by LF-NMR. In general, the areas of the PSD of different rocks vary, and the main distribution areas show single, double, and triple peaks, which are recognized as the typical distribution patterns of sandstone bodies. The PSD of the rocks of different lithologies was specifically analyzed. Fig 8(a) shows the PSD of coarse sandstone. C1–C4 are unweathered coarse sandstone, the pore distribution mostly presents a triple-peak pattern, and the left, middle, and right peaks are interconnected with little difference in the peak value, indicating that the connectivity of pores with different radii is relatively good. C5–C8 are weathered coarse sandstone, most of the pore distribution shows a right-skewed double-peak pattern, and the right peak shows an obvious shift toward macropores, indicating that weathered has changed the distribution and connectivity of the pores inside the coarse sandstone, and macropores are dominant at this stage. Fig 8(b) shows the PSD of medium sandstone. M1–M4 are unweathered medium sandstone, and the pore distribution mostly shows a double-peak pattern. M5–M8 are weathered medium sandstone, the pore distribution mostly shows a right-skewed double-peak pattern, and the right peak shows an obvious shift toward mesopores, indicating that weathered has changed the distribution and connectivity of the pores inside the medium sandstone, and mesopores are dominant at this stage. The PSD of fine the sandstone is shown in Fig 8(c). F1–F4 are unweathered fine sandstone, and the pore distribution mostly presents a single-peak pattern. F5–F8 are weathered fine sandstone, the pore distribution mostly shows a right-skewed double-peak pattern, and the right peak shows a significant shift toward mesopores, indicating that weathered has changed the distribution and connectivity of pores inside the fine sandstone, and mesopores are dominant at this stage. The PSD of unweathered siltstone and unweathered siltstone is shown in Fig 8(d). S1–S4 are unweathered siltstone, and S5–S8 are weathered siltstone. The pore distribution mostly shows a left-skewed triple-peak pattern, and the peaks are mainly concentrated near micropores and fine pores, with obvious boundaries being between the triple peaks and even between the zero-value areas. The poor continuity of the peaks reflects the poor connectivity of pores, and fine pores and micropores dominate inside the unweathered siltstone and weathered siltstone. The pore distribution of unweathered siltstone and weathered siltstone is essentially identical, with fine pores and micropores representing the predominant internal structure of both unweathered and weathered siltstone.

**Fig 9:**
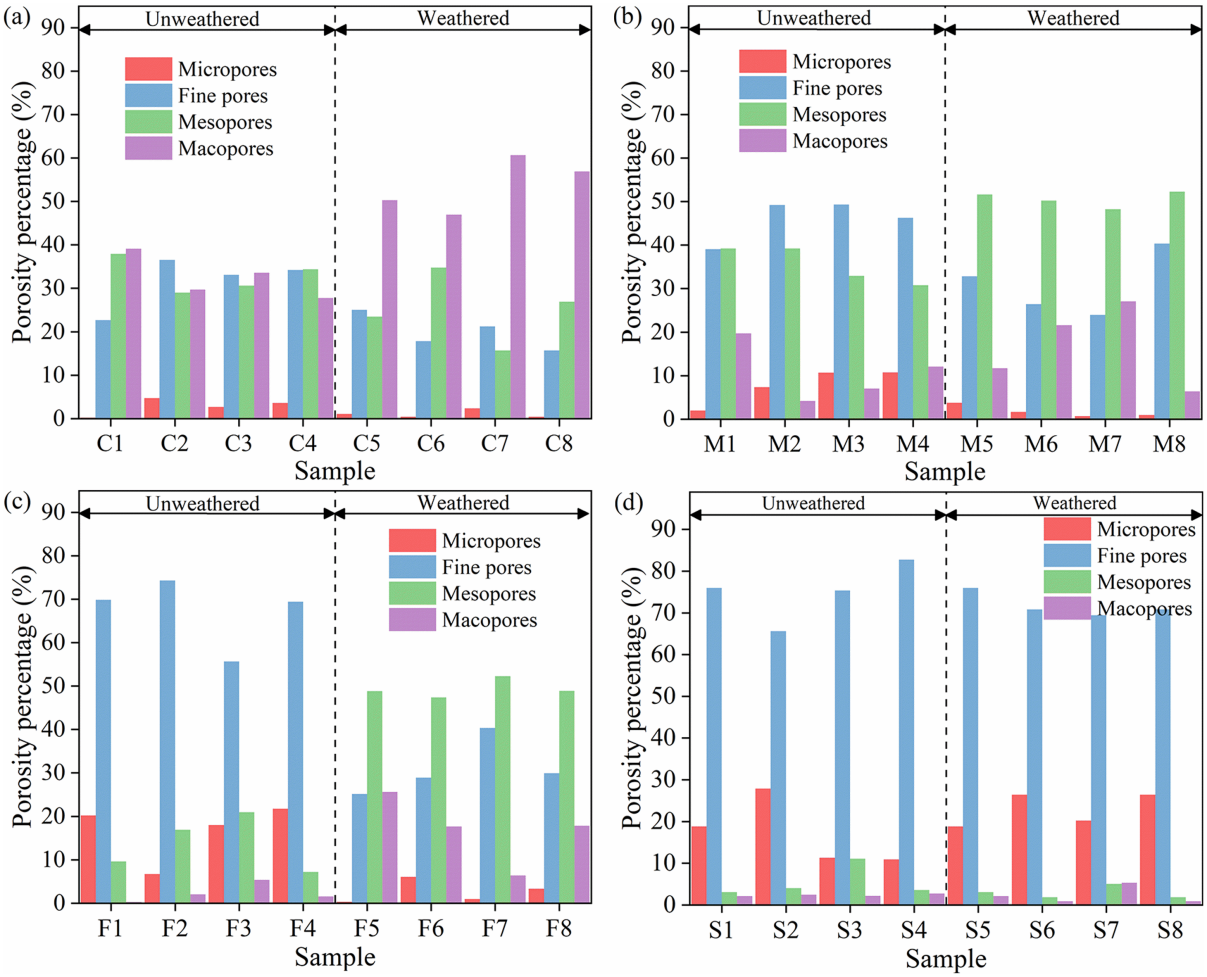
Pore volume distribution of different pore sizes in each lithology. (a) Coarse sandstone; (b) medium sandstone; (c) fine sandstone; (d) siltstone.

According to the evaluation criteria of PSD, the porosity of a porous structure can be calculated by the percentage of different micropore pore sizes. It is clear that there are variations in the pore structure caused by different lithologies and weathered. Fig 9 shows the percentage of pore volume for different rocks.

Fig 9(a) shows the percentage porosity for different pore sizes in weathered coarse sandstone and unweathered coarse sandstone. The average percentage of pore volume from micropores to macropores in the unweathered coarse sandstone are 2.78%, 31.66%, 33.01%, and 32.55%, respectively, and the pore volume percentages of fine pores, mesopores, and macropores are less different; The average pore volume percentages of micropores to macropores in the weathered coarse sandstone are 1.06%, 19.97%, 25.24%, and 53.73%, respectively, and macropores are dominant. Fig 9(b) shows the percentage porosity for different pore sizes in weathered and unweathered medium sandstones. The average percentage of pore volume from micropores to macropores in the unweathered medium sandstone are 7.69%, 45.98%, 35.55%, and 10.77%, respectively. The average pore volume percentages of micropores to macropores in the weathered medium sandstone are 1.77%, 30.91%, 50.61%, and 16.71%, respectively, and mesopores are dominant. Fig 9(c) shows the percentage porosity for different pore sizes in weathered fine sandstone and unweathered fine sandstone. The average percentage of pore volume from micropores to macropores in the unweathered fine sandstone are 16.67%, 67.35%, 13.69%, and 2.29%, respectively, and fine pores are dominant. The average pore volume percentages of micropores to macropores in the weathered fine sandstone are 2.66%, 31.09%, 49.37%, and 16.88%, respectively, and mesopores are dominant. Fig 9(d) shows the percentage porosity for different pore sizes in unweathered siltstone and weathered siltstone. The average percentage of pore volume from micropores to macropores in the unweathered siltstone are 17.25%, 74.95%, 5.43%, and 2.37%, respectively. The average percentage porosity from micropores to macropores in the weathered siltstone are 22.97%, 71.79%, 2.95%, and 2.29%, respectively. The pore volume percentages of different pore sizes in unweathered siltstone and weathered siltstone are less different, and both types of rocks are dominated by fine pores and micropores.

The pore structure of the rock of the same lithology varied after being subjected to weathered, with an overall trend of shifting from small to large pore sizes. This results in a variation in the water-bearing properties of the sandstone, with the weathered bedrock of the sandstone having a higher water-bearing properties than the corresponding unweathered bedrock.

### 3.2 Permeability characteristics of LF-NMR

The semidefinite relaxation (SDR) model are an effective method for estimating the permeability of sandstone [40]. The SDR model for estimating the permeability can be calculated by Eq (4).


$$K_{nmr}=\lambda \times T_{m_{1}}^{2lm}\times \varphi^{n_{1}}$$ (4)

where K_nmr_ is the estimated penetration rate, and its unit is mD; λ, m_1_ and n_1_ are the corresponding fitting parameters. The values of λ, m1 and n1 were chosen from the suggested parameter values of previous studies, which were 10, 2 and 4 respectively [41].

The porosity of 32 groups of rocks was determined using NMR measurements. Based on this, the permeability of the rocks of different lithologies was separately calculated using Eq. (4). Table 3 shows the results of the porosity and permeability calculations.

**Table 3:** Porosity and permeability of different types of rocks.

Rock type	Sample	Weather or not	Porosity /%	Average porosity /%	Standard deviation	Permeability /md	Average permeability/md	Standard deviation
Siltstone	S1	Unweathered	5.98	6.50	0.4445	0.0003	0.0002	0.0001
	S2	Unweathered	7.18			0.0004		
	S3	Unweathered	6.27			0.0003		
	S4	Unweathered	6.57			0.0001		
	S5	Weathered	7.56	6.78	0.4880	0.0003	0.0003	0.00005
	S6	Weathered	6.49			0.0003		
	S7	Weathered	6.79			0.0004		
	S8	Weathered	6.27			0.0004		
Fine sandstone	F1	Unweathered	8.00	7.77	0.5450	0.0012	0.0009	0.0003
	F2	Unweathered	7.02			0.0006		
	F3	Unweathered	8.50			0.0013		
	F4	Unweathered	7.57			0.0006		
	F5	Weathered	8.65	8.64	0.2897	0.0026	0.0029	0.0006
	F6	Weathered	8.82			0.0033		
	F7	Weathered	8.16			0.0020		
	F8	Weathered	8.91			0.0036		
Medium sandstone	M1	Unweathered	9.71	9.45	0.2782	0.0032	0.0026	0.0004
	M2	Unweathered	9.72			0.0027		
	M3	Unweathered	9.32			0.0021		
	M4	Unweathered	9.06			0.0023		
	M5	Weathered	10.32	10.30	0.1914	0.0046	0.0044	0.0005
	M6	Weathered	10.09			0.0037		
	M7	Weathered	10.19			0.0042		
	M8	Weathered	10.60			0.0051		
Coarse sandstone	C1	Unweathered	11.54	11.71	0.1901	0.0079	0.0072	0.0011
	C2	Unweathered	11.57			0.0066		
	C3	Unweathered	11.72			0.0086		
	C4	Unweathered	12.02			0.0059		
	C5	Weathered	13.22	13.05	0.4601	0.0161	0.0135	0.0023
	C6	Weathered	13.72			0.0156		
	C7	Weathered	12.72			0.0104		
	C8	Weathered	12.54			0.0120		

**Fig 10:**
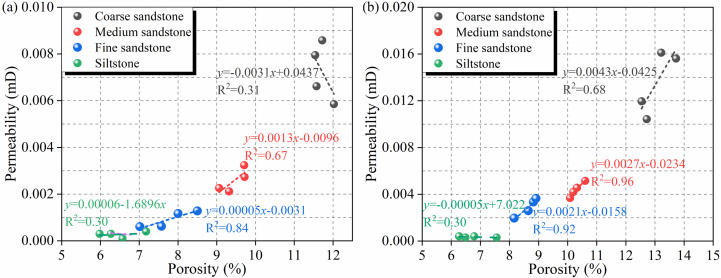
Total porosity versus permeability of the rocks of different lithologies in the study area. (a) Unweathered bedrock; (b) weathered bedrock.

Table 2 and Fig 10 show the correlation between total porosity and permeability for different rocks. Particle sizes and weathered dramatically affect the total porosity and permeability of sandstones. Fig 10(a) shows the correlation between the total porosity and permeability of the different unweathered rock masses. It is found that the permeability of coarse sandstone is negative correlated with the total porosity, and other rock types have a positive correlation between permeability and total porosity. Fig 10(b) shows the correlation between the total porosity and permeability of different rock masses after weathered. It is found that the permeability of siltstone is negative correlated with the total porosity, and other rock types have a positive correlation between permeability and total porosity.

In conjunction with the pore distribution analyses carried out in the previous stage, it can be discerned that the shift from a negative to a positive correlation can be ascribed to the augmented distribution of mesopores and macropores arising from weathered. This expansion of mesopores and macropores boosts the connectivity among pores. The negative correlation witnessed in siltstone can be attributed to the circumstance that the porosity of this rock type is relatively low both before and after weathered. Moreover, the predominant pore size range remains micropores and fine pores. While weathered does induce an increase in porosity, this is mainly due to an expansion in the number of micropores and fine pores, which in turn leads to poor connectivity between siltstone pores.

**Fig 11:**
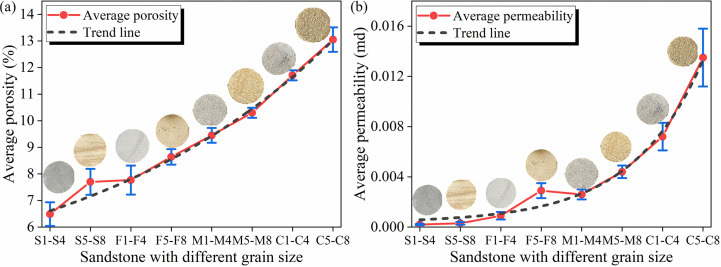
Relationships of different sandstones with the average porosity and average permeability in the study area. (a) Average porosity; (b) average permeability.

Permeability are shown in Table 2 and Fig 11. Fig 11(a) shows the relationships between different sandstones and the average porosity. It was found that the porosity of different sandstones was positively correlated with rock particle size, and the porosity of the rock also increases under weathered. For example, for unweathered rocks of different lithologies, the average porosity of the siltstone to coarse sandstone is 6.49 ± 0.4445%, 7.77 ± 0.545%, 9.45 ± 0.1932%, and 11.71 ± 0.1901% (mean ± standard deviation (SD)), respectively. For weathered rocks of different lithologies, the average porosity of the siltstone to coarse sandstone is 7.7 ± 0.488%, 8.64 ± 0.2897%, 10.3 ± 0.1914%, and 13.05 ± 0.4601%, respectively. Fig 11(b) shows the relationships between different lithologies and the average permeability. The permeability of the rocks all increase with increasing grain size, and the permeability of the rock of the same lithology also increases under the weathered action. The two reasons for the above phenomenon may be as follows. One is that the size of intergranular pores decreases with a decrease in grain size. The other is that weathered causes the elements in the sandstone to become sparse surrounded by minerals, leading to an increase in porosity and corresponding permeability.

In general, there are good correlation between the porosity and permeability of rocks, the relationships between the average permeability and average total porosity of different types of unweathered sandstones and weathered sandstones are basically not significantly different; the effects of grain sizes and weathered of lithologies on the total porosity are basically consistent with their effects on the permeability. These phenomena indicate that the primary fractures in the strata of the study area are less developed, and the permeability of the strata is mainly influenced by the microscopic pore structures in the strata. Water is mainly distributed on the surfaces of these micropores, which are an important part of dense sandstones. The microporous structures are significant for the permeability of aquifers with different properties.

### 3.3 Multifractal characteristics of LF-NMR

#### 3.3.1 Multifractal model for LF-NMR.

The fractal characteristics of rock micropore structures with different pore sizes are obviously different, and the fractal dimension can be used to quantitatively describe the complexity of rock pore structure [42]. Normally a larger fractal dimension indicates a more complex pore structure and greater pore heterogeneity in the rock [43].

The number of rock pore radius greater than *r*. The relationship between mesh N and pore radius r satisfies a power function, such as Eq. (5).


$$N(�r)=\int_{r}^{r_{\max}}P(r)dr=\alpha \times r^{-D}$$ (5)

where *r*_*max*_ is the largest pore radius, *P* (*r*) is the density function of pore size distribution, *a* is constant, and *D* is fractal size.

The cumulative pore volume *V*_*P*_ for LF-NMR is calculated by Eq. (6).


$$V_{P}={\frac{T_{2\max}}{T_{2}}}^{D-3}$$ (6)

Eq. (7) can be obtained by taking logarithms on both sides of Eq. (6).


$$\mathrm{lg}(V_{P})=(3-D)\mathrm{lg}\left(T_{2}/T_{2\max}\right)$$ (7)

Based on the NMR parameters and Eq. (7), D can be calculated by Eq. (8).


D=3−K (8)

where *K* is the slope of log (*V*_*P*_) to Lg(T_2_/T_2*max*_).

#### 3.3.2 Multifractal dimension of LF-NMR.

**Fig 12:**
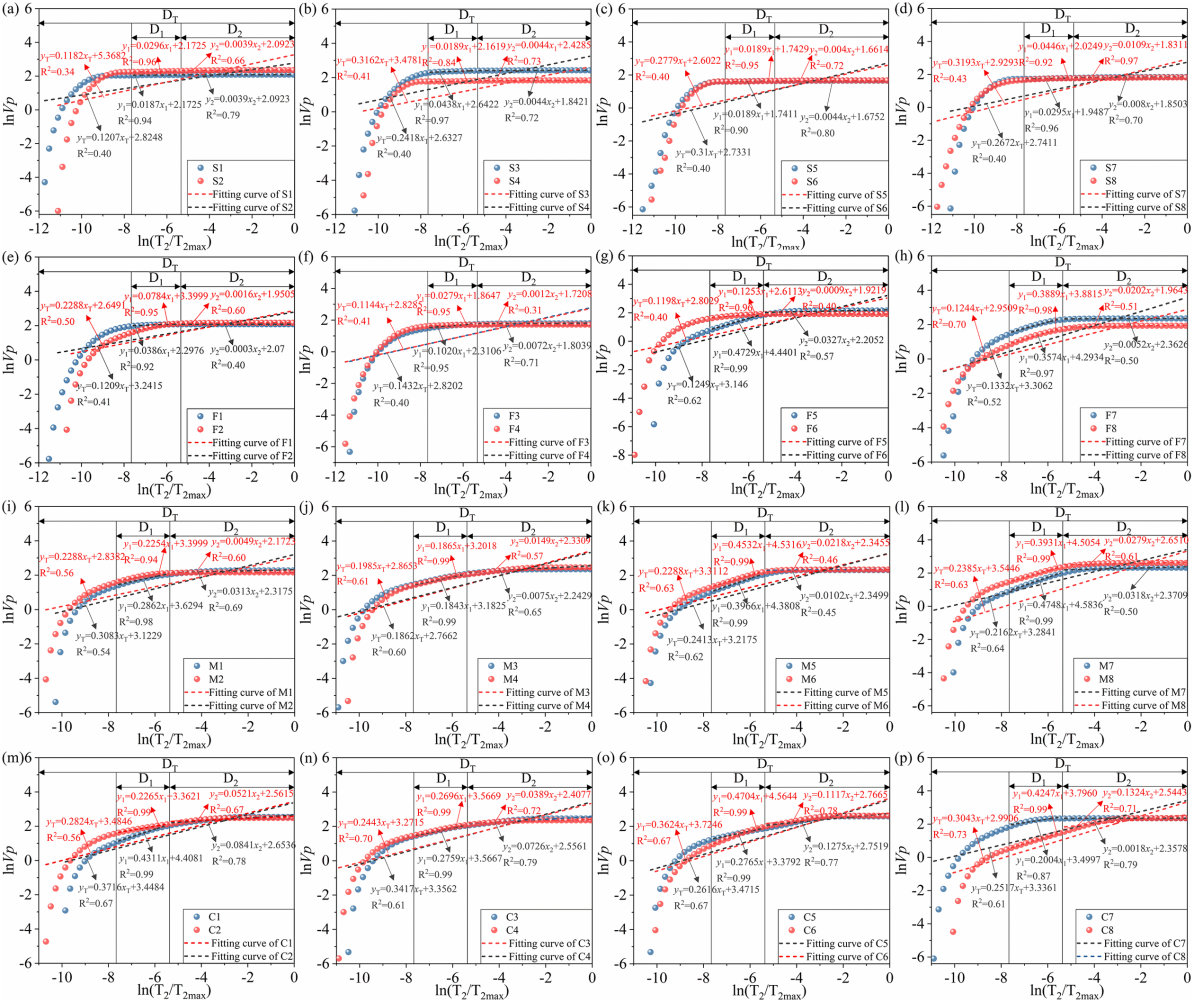
Fractal dimension fitted curves for different sandstones. (a–d) Coarse sandstone; (e–h) medium sandstone; (i–l) fine sandstone; (m–p) siltstone.

Based on the fractal model for LF-NMR, 32 sets of fractal dimension fitted curves for different rocks can be obtained according to Eqs (7) and (8), and the corresponding fractal dimensions can be obtained through computational analysis. Fig 12 shows the NMR fractal dimension fitted curves for different rocks. When plotting the log (V_P_)~log(T_2_/T_2max_) distribution based on NMR parameters and the fractal theory, the fractal dimension of this region is < 2 due to the large overall slopes of the micropore and fine pore sections. The fractal dimension is not in accordance with the definition of the rock fractal, so it is not of practical significance, and no specific analysis of the fractal dimensions of micropores and fine pores was done in this case.

The data can be divided into three parts according to the distribution characteristics of different rocks. After linear fitting of these three parts, the dashed part with the larger slope indicates the fractal dimension of the medium hole (D_1_), the dashed part with the smaller slope indicates the fractal dimension of the large hole (D_2_) and the solid part with the larger slope indicates the fractal dimension of the storage unit (D_T_). The slopes of the curves decrease as the pore diameters and volumes increase, the water content in the pores increases, and the responses to the applied magnetic field become slower from small pores to large pores. Table 4 shows the specific calculation results for the different fractals.

**Table 4:** Fractal dimensions of different rock samples.

Sample	NMR regression equation	D_1_	NMR regression equation	D_2_	NMR regression equation	D_T_
S1	*y*= 0.0187*x* + 2.1725 R^2^=0.94	2.9813	*y*= 0.0039*x +* 2.0923 R^2^=0.79	2.9961	*y*= 0.1207*x +* 2.8248 R^2^=0.40	2.8793
S2	*y*= 0.0296*x* + 4.7150 R^2^=0.96	2.9704	*y*= 0.0076*x +* 4.6158 R^2^=0.66	2.9924	*y*= 0.1182*x +* 5.3682 R^2^=0.34	2.8818
S3	*y*= 0.0438*x* + 2.6422 R^2^=0.97	2.9562	*y*= 0.0044*x +* 1.8421 R^2^=0.72	2.9956	*y*= 0.2418*x +* 2.6327 R^2^=0.40	2.7582
S4	*y*= 0.0189*x* + 2.1619 R^2^=0.84	2.9811	*y*= 0.0044*x +* 2.4285 R^2^=0.73	2.9956	*y*= 0.3162*x +* 3.4781 R^2^=0.41	2.6838
S5	*y*= 0.0189*x* + 1.7411 R^2^=0.90	2.9801	*y*= 0.0044*x +* 1.6752 R^2^=0.80	2.9956	*y*= 0.3100*x +* 2.7331 R^2^=0.40	2.6900
S6	*y*= 0.0189*x* + 1.7429 R^2^=0.95	2.9801	*y*= 0.004*x +* 1.6614 R^2^=0.72	2.9960	*y*= 0.2779*x +* 2.6022 R^2^=0.40	2.7221
S7	*y*= 0.0295*x* + 1.9487 R^2^=0.96	2.9705	*y*= 0.008*x +* 1.8503 R^2^=0.70	2.9920	*y*= 0.2672*x +* 3.1213 R^2^=0.40	2.7328
S8	*y*= 0.0446*x* + 2.0249 R^2^=0.92	2.9554	*y*= 0.0109*x +* 1.8311 R^2^=0.97	2.9891	*y*= 0.3193^+^ 2.9293 R^2^=0.43	2.6807
F1	*y*= 0.0386*x* + 2.2976 R^2^=0.92	2.9614	*y*= 0.0003*x +* 2.0799 R^2^=0.40	2.9997	*y*= 0.1209*x +* 3.2415 R^2^=0.41	2.8791
F2	*y*= 0.0784*x* + 3.3999 R^2^=0.95	2.7746	*y*= 0.0016*x +* 1.9505 R^2^=0.57	2.9984	*y*= 0.2288*x +* 2.6491 R^2^=0.50	2.7712
F3	*y*= 0.1020*x* + 2.3106 R^2^=0.95	2.8980	*y*= 0.0072*x +* 1.8039 R^2^=0.71	2.9928	*y*= 0.1432*x +* 2.8202 R^2^=0.40	2.8568
F4	*y*= 0.0279*x* + 1.8647 R^2^=0.95	2.9721	*y*= 0.0012*x +* 1.7202 R^2^=0.31	2.9988	*y*= 0.1144*x +* 2.8285 R^2^=0.41	2.8856
F5	*y*= 0.4729*x* + 4.4401 R^2^=0.99	2.5271	*y*= 0.0327*x +* 2.2052 R^2^=0.57	2.6724	*y*= 0.1249*x +* 3.1460 R^2^=0.62	2.8751
F6	*y*= 0.1253*x* + 2.6113 R^2^=0.96	2.8747	*y*= 0.0009*x +* 1.9219 R^2^=0.35	2.9989	*y*= 0.1198*x +* 2.8029 R^2^=0.40	2.8802
F7	*y*= 0.3574*x* + 4.2834 R^2^=0.97	2.6426	*y*= 0.0052*x +* 2.3626 R^2^=0.50	2.9948	*y*= 0.1332*x +* 3.3062 R^2^=0.52	2.8668
F8	*y*= 0.3889*x* + 3.8815 R^2^=0.98	2.6111	*y*= 0.0202*x +* 1.9643 R^2^=0.50	2.9798	*y*= 0.1244*x +* 2.9509 R^2^=0.62	2.8756
M1	*y*= 0.2862*x* + 3.6294 R^2^=0.98	2.7138	*y*= 0.0313*x +* 2.3175 R^2^=0.69	2.9687	*y*= 0.3083*x +* 3.1229 R^2^=0.54	2.6917
M2	*y*= 0.2254*x* + 3.3999 R^2^=0.94	2.7746	*y*= 0.0049*x +* 2.1723 R^2^=0.60	2.9951	*y*= 0.2288*x +* 2.8382 R^2^=0.56	2.7712
M3	*y*= 0.1843*x* + 3.1825 R^2^=0.99	2.8157	*y*= 0.0075*x +* 2.2429 R^2^=0.65	2.9925	*y*= 0.1862*x +* 2.7662 R^2^=0.60	2.8138
M4	*y*= 0.1865*x* + 3.2018 R^2^=0.99	2.8135	*y*= 0.0149*x +* 2.3309 R^2^=0.57	2.9851	*y*= 0.1985*x +* 2.8653 R^2^=0.61	2.8015
M5	*y*= 0.3966*x* + 4.3808 R^2^=0.99	2.6034	*y*= 0.0102*x +* 2.3499 R^2^=0.45	2.9898	*y*= 0.2413*x +* 3.2175 R^2^=0.62	2.7587
M6	*y*= 0.4532*x* + 4.5316 R^2^=0.99	2.5468	*y*= 0.0218*x +* 2.3455 R^2^=0.44	2.9782	*y*= 0.2288*x +* 3.3112 R^2^=0.6229	2.7712
M7	*y*= 0.4748*x* + 4.5836 R^2^=0.99	2.5252	*y*= 0.0318*x +* 2.3709 R^2^=0.50	2.9682	*y*= 0.2162*x +* 3.2841 R^2^=0.64	2.7838
M8	*y*= 0.3931*x* + 4.5054 R^2^=0.99	2.6069	*y*= 0.0279*x +* 2.6510 R^2^=0.61	2.9721	*y*= 0.2385*x +* 3.5446 R^2^=0.63	2.7615
C1	*y*= 0.4311*x* + 4.4081 R^2^=0.99	2.5689	*y*= 0.0841*x +* 2.6536 R^2^=0.78	2.9159	*y*=0.3716*x +* 3.4484 R^2^ = 0.67	2.6284
C2	*y*= 0.2265*x* + 3.3621 R^2^=0.99	2.7735	*y*= 0.0521*x +* 2.5615 R^2^=0.67	2.9479	*y*=0.2824*x +* 3.4846 R^2^ = 0.56	2.7176
C3	*y*= 0.2759*x* + 3.5667 R^2^=0.99	2.7241	*y*= 0.0726*x +* 2.5561 R^2^=0.79	2.9274	*y*=0.3417*x +* 3.3562 R^2^ = 0.61	2.6583
C4	*y*= 0.2696*x +* 3.5669 R^2^=0.99	2.7304	*y*= 0.0389*x +* 2.4077 R^2^=0.72	2.9611	*y*=0.2443*x +* 3.2715 R^2^ = 0.70	2.7557
C5	*y*= 0.2765*x +* 3.3792 R^2^=0.99	2.7235	*y*= 0.1275*x +* 2.7519 R^2^=0.77	2.8725	*y*=0.2616*x +* 3.4715 R^2^ = 0.	2.7384
C6	*y*= 0.4704*x +* 4.5644 R^2^=0.99	2.5297	*y*= 0.1117*x +* 2.7665 R^2^=0.78	2.8883	*y*=0.3624*x +* 3.7246 R^2^ = 0.67	2.6376
C7	*y*= 0.2004*x +* 3.4997 R^2^=0.87	2.7996	*y*= 0.0018*x +* 2.3578 R^2^=0.79	2.9982	*y*=0.2517*x +* 3.3361 R^2^ = 0.61	2.7483
C8	*y*= 0.4247*x +* 3.7960 R^2^=0.99	2.5753	*y*= 0.1324*x +* 2.5443 R^2^=0.71	2.8676	*y*=0.3043*x +* 2.9906 R^2^ = 0.73	2.6957

**Fig 13:**
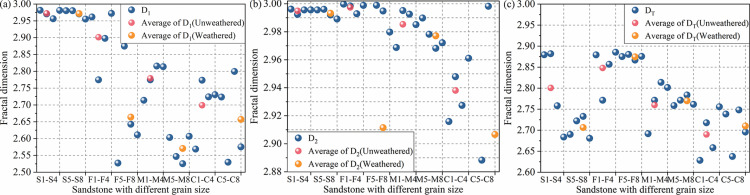
Fractal dimensions of different sandstones. (a) D_1_; (b) D_2_; (c) D_T_.

Fig 13 shows the trends of the average fractal dimensions of rocks of different lithologies. The overall trends of D_1_, D_2_, and D_T_ decrease with an increase in grain size, with good similarity. The fractal dimension of mesopores and macropores of the same type of sandstone decreased after weathered, which indicates that the complexity of the pore structure of mesopores and macropores in the sandstone body decreased after weathered. However, by comparing the fractal values of the overall pore structure, it can be seen that the fractal dimension of the overall pore structure of the sandstones, except for the siltstone, increased after weathered, indicating that weathered can cause the pore structure of the sandstones to become more complex in general. The main reason for the decrease of fractal dimension of siltstone is that the pore structure of siltstone is still dominated by micropores and fine pores after weathered, and the increase of micropores and fine pores caused by weathered has led to the improvement of the connectivity between the corresponding pores.

#### 3.3.3 Influence of rock types and weathered on multifractal characteristics.

To explore the relationship between D and pore size percentages for different rocks, linear fitting was carried out on the fractal dimension and pore percentage of unweathered and weathered rocks.

**Fig 14:**
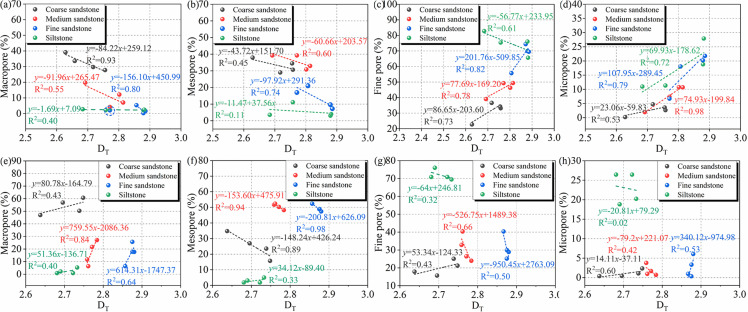
Fitted curves of D_T_ and the percentages of different sizes of pores in unweathered sandstones and weathered sandstones. (a–d) Unweathered; (e~h) weathered.

Fig 14 shows the fitted curves of D_T_ and the percentages of different sizes of pores in unweathered sandstones and weathered sandstones. As shown in Fig 14(a), the percentage of macropores from siltstone to coarse sandstone is negatively correlated with D_T_. As shown in Fig 14(b), the percentage of mesopores from siltstone to coarse sandstone is negatively correlated with D_T_. As shown in Fig 14(c), the percentage of fine pores in coarse sandstone to fine sandstone is positively correlated with D_T_, while the percentage of fine pores in siltstone is negatively correlated with D_T_. As shown in Fig 14(d), the percentage of macropores from siltstone to coarse sandstone is positively correlated with D_T_. As shown in Fig 14(e), the percentages of macropores in the coarse sandstone to siltstone are positively correlated with D_T_. As shown in Fig 14(f), the percentage of fine pores in coarse sandstone to fine sandstone is negatively correlated with D_T_, while the percentage of fine pores in siltstone is positively correlated with D_T_. As shown in Fig 14(g), the percentage of fine pores in the coarse sandstone is positively correlated with D_T_, and the percentage of fine pores in the other sandstones is negatively correlated with D_T_. As shown in Fig 14(h), the percentage of micropores in the medium sandstone and siltstone is negatively correlated with D_T_, and the percentage of micropores in the coarse sandstone and fine sandstones is positively correlated with D_T_.

The above results show that for different types of unweathered sandstone, the greater the proportion of micropores and micropores, the greater the fractal dimension and the more complex the pore structure, mainly because micropores and micropores have smaller pore sizes and volumes, separating the rock into more tiny spaces, increasing the complexity of the pore structure and reducing the connectivity between pores, leading to an increase in the fractal dimension. Weathered caused large changes in the pore structures of coarse sandstone to fine sandstone; For different types of sandstone after weathered, larger percentages of macropores indicated larger fractal dimensions and more complex pore structures. This phenomenon is mainly due to the deterioration of the connectivity of macropores due to weathered, which leads to the increase of fractal size. This study showed that the connectivity of the pore structures of different rock masses depended on the coexistence of different pore sizes, but the fine pores and micropores might account for a large proportion of the total pores, the connectivity of the pore structure in weathered sandstone is mainly determined by the distribution of pore systems with large pore sizes.

**Fig 15:**
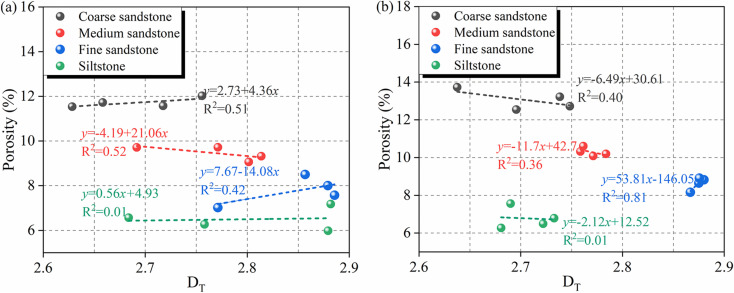
Fitted curves of D_T_ and the total porosity of rocks of different lithologies. (a) Unweathered rocks; (b) weathered rocks.

To explore the correlations of the total fractal with and the total porosity, linear fitting was performed on the fractal dimensions and total porosity (Fig 15). Fig 15 shows the fitted curves of D_T_ and the total porosity of rocks of different lithologies. Fig 15(a) displays that the total porosity of unweathered coarse sandstone, fine sandstone, and sandy mudstone is positively correlated with D_T_, while the total porosity of the medium sandstone and siltstone is negatively correlated with D_T_. Fig 15(b) displays that the total porosity of the weathered coarse sandstone, medium sandstone and siltstone is negatively correlated with D_T_, and the total porosity of the fine sandstone is positively correlated with D_T_. The above results showed that the correlations between the total porosity and D_T_ of different rocks varied, and the rock types and weathered affected the microscopic pore structures of rock strata; porous media with the same D_T_ or porosity could have different microporous structures; Weathered changing the correlation between the total porosity and DT of the coarse sandstone from positive to negative.

This phenomenon indicated that weathered caused better connectivity of micropores of different pore sizes in coarse sandstone, contributing to groundwater storage and transport. The fractal dimension of siltstone in the sample is weakly correlated with different types of pores, indicating that the fractal dimension of sandstone is mainly influenced by mesopores and macropores, in other words, the complexity between pores in sandstone is mainly influenced by mesopores and macropores.

#### 3.3.4 Relationship between the fractal dimension and mineral composition.

**Fig 16:**
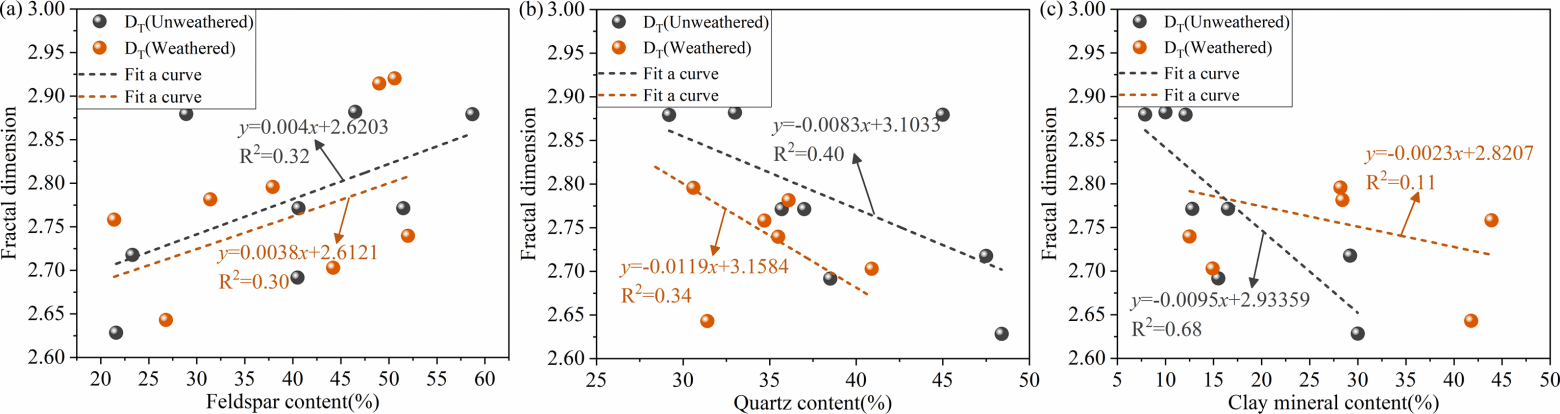
Correlation between the sandstone mineral composition and fractal dimension. (a) Feldspar content; (b) quartz content; (c) clay mineral content.

Fig 16 shows the relationship between the fractal dimension and quartz, feldspar, and clay minerals contents in the study area. It can be seen that the feldspar content in the study area shows a positive correlation with the total fractal of the pores. Quartz and clay mineral contents show a negative correlation with the total fractal of the pores. The dissolution pore space in dense sandstone increases with the elevation in feldspar content. When feldspar grains dissolve, the resulting internal pore spaces predominantly display harbour- or banshee-like configurations. Moreover, they are poorly connected, increasing the complexity of the pore network. Quartz contributes to rocks’ physical stability and can enhance rock layers’ compaction resistance. This facilitates the preservation of primary pores and reduces permeability and pore loss. Conversely, reducing the pore number allows for easy pore connectivity and lowers the fractal dimension of the sandstone. Quartz plays a vital role in the structural formation of dense sandstone. A higher content of quartz contributes to a stronger compaction resistance of sandstone. In addition, a larger size of intergranular pores indicates better connectivity. This improvement can reduce the non-homogeneity and complexity of pores. Throughout long-term weathered, the deformation and filling of clay minerals degrade the pore connectivity. As a result, the pore structural complexity and the fractal dimension of sandstone are reduced. Based on the above analysis, weathered and mineral composition primarily affect the pore fractal dimension of sandstone. The correlation between mineral content and fractal dimension also varies across different sandstones.

### 3.4 Influence of weathered on porosity evolution

**Fig 17:**
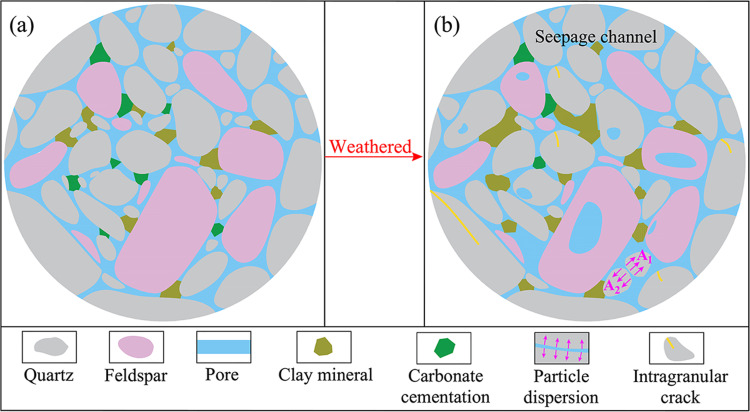
Effect mechanism of weathered on porosity of sandstone.

The mechanism model illustrating the influence of weathered on sandstone porosity is depicted in Fig 17. Sandstone undergoes changes due to variations in the mechanical strength and cementation degree among different mineral grains during weathered, resulting in various degrees of susceptibility to this condition. Based on the derived findings, unweathered and weathered sandstones primarily consist of quartz, feldspar, dolomite, and clay mineral crystals, exhibiting varying-sized pores. Physical and chemical weathered processes are mainly responsible for the change in weathered sandstone porosity. Physical weathered results in mechanical disintegration and fracture of rocks, and these fragments accumulate as loose deposits. When this process further progresses, the rock fissure degree intensifies, the permeability increases, the weathered debris exhibits decreased diameter, and the surface area of particles increases. These alterations provide favourable conditions for the occurrence of chemical weathered. These micropores were enlarged by erosion and developed into microcracks, which enable salt/acid-rich fluids to penetrate the sandstone and facilitate subsequent hydration and crystallization. The carbon dioxide in the water reacts chemically with rock minerals. Due to this reaction, some feldspar and cements dissolve and form soluble minerals. The water washes away these soluble minerals, and the less soluble clay minerals persist. In addition, dissolved substances in water undergo displacement reactions with minerals within rocks, forming clay minerals.

Considerable feldspars and other knots gradually consume the organic acid released in the previous stage. During this process, the pH value of the pore fluid increases, contributing to the dissolution of quartz. Moreover, some of the tiny quartz crystals disintegrate under weathered, destroying the connection between grains, which then break up into multiple particles, facilitating the formation of a pore channel. For example, grain A is decomposed into two separate particles, namely A_1_ and A_2_. The formation of clay minerals is typically driven by the transformation of the original rock due to weathered.

## 4 Conclusions

In this study, the commonalities and differences of the pore structures and characteristics of different rocks before and after weathered were investigated using LF-NMR. Based on this, the effects of weathered on the permeability of different rocks were analyzed, and the relationships of the fractal dimensions and the percentage with each pore size and the total porosity of different rocks were explored. Based on the experimental data obtained in this study and comprehensive analysis, the following conclusions were obtained.

(1) The XRD of sandstone samples with different weathered degrees is basically consistent. This observation indicates that no changes occur in the main minerals in sandstone with different grain sizes due to weathered. After weathered, the content of quartz and feldspar in sandstone decreases, and the content of clay minerals increases gradually. Among them, the clay mineral content of coarse sandstone is more obvious than that of other sandstones. The mineral content of coarse sandstone increases by 13.25%.
(2) The total porosity of different rocks generally increased with an increase in rock grain size and the pore structure of the same rock changed from small- to large-pore type overall after weathered. The unweathered coarse sandstone was dominated by fine pores, mesopores, and macropores with small differences, and the weathered coarse sandstone was dominated by macropores; the unweathered medium and fine sandstones were dominated by fine pores, and the weathered medium and fine sandstones were dominated by mesopores; the unweathered siltstone and weathered siltstone were both dominated by fine pores and micropores.
(3) For different rocks, greater proportions of micropores and fine pores before weathered and a greater proportion of macropores after weathered led to larger corresponding fractal dimensions of the rocks. The results indicated that although the the connectivity of the pore structures of different rocks depended on the coexistence of macropores, fine pores, and micropores, the connectivity of the pore structures of different rocks after weathered were mainly determined by the distribution of pore systems with large pore sizes.
(4) The fractal dimensions D_1_, D_2_, and D_T_ of different rocks showed overall decreasing trends with increases in particle sizes. The rocks with the same D_T_ or porosity could have different microporous structures, and weathered affected the fractal characteristics of the microscopic pore structures of rocks. The rock with the greatest effect of weathered on the change in the connectivity of the pore structure was the coarse sandstone, causing the relationship between total porosity and D_T_ in the coarse sandstone to change from a positive to a negative correlation, indicating that weathered resulted in better connectivity of micropores of different pore sizes in the coarse sandstone.

## Supporting information

S1 Data Data. (ZIP)
